# Nectar compounds impact bacterial and fungal growth and shift community dynamics in a nectar analog

**DOI:** 10.1111/1758-2229.13139

**Published:** 2023-02-13

**Authors:** Tobias G. Mueller, Jacob S. Francis, Rachel L. Vannette

**Affiliations:** ^1^ Department of Entomology and Nematology University of California, Davis Davis California USA; ^2^ Department of Entomology Cornell University Ithaca New York USA

## Abstract

Floral nectar is frequently colonised by microbes. However, nectar microbial communities are typically species‐poor and dominated by few cosmopolitan genera. One hypothesis is that nectar constituents may act as environmental filters. We tested how five non‐sugar nectar compounds as well as elevated sugar impacted the growth of 12 fungal and bacterial species isolated from nectar, pollinators, and the environment. We hypothesised that nectar isolated microbes would have the least growth suppression. Additionally, to test if nectar compounds could affect the outcome of competition between microbes, we grew a subset of microbes in co‐culture across a subset of treatments. We found that some compounds such as H_2_O_2_ suppressed microbial growth across many but not all microbes tested. Other compounds were more specialised in the microbes they impacted. As hypothesised, the nectar specialist yeast *Metschnikowia reukaufii* was unaffected by most nectar compounds assayed. However, many non‐nectar specialist microbes remained unaffected by nectar compounds thought to reduce microbial growth. Our results show that nectar chemistry can influence microbial communities but that microbe‐specific responses to nectar compounds are common. Nectar chemistry also affected the outcome of species interactions among microbial taxa, suggesting that non‐sugar compounds can affect microbial community assembly in flowers.

## INTRODUCTION

Most angiosperms produce floral nectar to attract pollinators. Floral nectar (hereafter simply nectar) is an aqueous solution often predominantly composed of sugars including sucrose, glucose, and fructose (Baker & Baker, 1983). However, nectar is much more than a simple sugar solution; approximately 10% of nectar's dry weight is composed of non‐sugar compounds including free amino acids, proteins, lipids, vitamins, and alkaloids among other compounds (Baker, 1977; Nicolson et al., 2007; Roy et al., 2017) and can differ substantially among and within species (Nicolson et al., 2007; Ryniewicz et al., 2020).

Nectar can be colonised by microbes, primarily yeasts and bacteria, which are deposited by floral visitors (Russell et al., 2019; Sandhu & Waraich, 1985; Zemenick et al., 2021). Surveys typically find 20%–50% of flowers contain culturable microbes depending on plant species and environment (Álvarez‐Pérez et al., 2012; de Vega et al., 2009; Jacquemyn et al., 2013; Pozo et al., 2011; Vannette et al., 2021). The microbes found in nectar can range from plant and pollinator pathogens, to putatively mutualistic, to microbes that may be commensal or have no documented effects on plants or pollinators (Adler et al., 2021). Once deposited, nectar microbes can reach high densities, growing to more than 10^5^ cells/μl for yeasts and 10^7^ cells/μl for bacteria (Álvarez‐Pérez et al., 2019). However, microbial communities often exhibit low alpha diversity within individual nectar samples, consisting of a few globally dominant genera, including fungi, such as *Metschnikowia* and *Aureobasidium* (Chappell & Fukami, 2018; de Vega et al., 2009; Pozo et al., 2011), and bacteria, such as *Acinetobacter* (Álvarez‐Pérez et al., 2012; Alvarez‐Pérez & Herrera, 2013; Fridman et al., 2012; Tsuji & Fukami, 2018). The microbes that establish in nectar are a subset of the microbes carried by pollinators and in the environment (Alvarez‐Pérez & Herrera, 2013; Herrera et al., 2010; Pozo et al., 2012). While it is clear that many microbes deposited in floral nectar fail to establish (de Vega & Herrera, 2012; Herrera et al., 2010; Pozo et al., 2012), numerous processes may generate the low microbial diversity observed in nectar. Possible mechanisms include differential dispersal of microbes (Zemenick et al., 2021); competitive exclusion that favours early arriving, faster growing, or inhibiting species (Dhami et al., 2016; Fukami, 2015); or strong filtering by the chemistry of the nectar environment (Herrera et al., 2010). These mechanisms are not mutually exclusive and likely vary in importance depending upon context. However, in some systems animal‐flower visitation networks alone cannot explain nectar microbial communities suggesting that filtering may play a role (Zemenick et al., 2021).

Some nectar traits are thought to provide antimicrobial activity (Herrera et al., 2010; Schmitt et al., 2021). The high sugar concentrations in nectar leads to extreme osmotic pressure and high C:N ratios both of which limit microbial growth (Brysch‐Herzberg, 2004; Herrera et al., 2010; Lievens et al., 2015). Additionally, antimicrobial compounds are commonly produced in nectar (Schmitt et al., 2018, 2021). In ornamental tobacco (*Nicotiana langsdorffii* × *Nicotiana sanderae*), hydrogen peroxide levels can reach 4 mM (Carter & Thornburg, 2004), suppressing some but not all microbes' growth (Carter et al., 2007; Parra et al., 2022). Other antimicrobial proteins are thought to have activity against specific groups of microbes (Schmitt et al., 2021). In previous comparative studies, nectar compounds including hydrogen peroxide, the antimicrobial protein BrLTP2.1, and the floral volatile linalool showed species‐specific effects, reducing microbial growth for some species but not others (Block et al., 2019; Burdon et al., 2018; Carter et al., 2007; Schmitt et al., 2018). However, few studies have broadly compared if microbes isolated from nectar and other habitats, vary in resistance to a range of nectar compounds (however, see Burdon et al., 2018; Mittelbach et al., 2016; Pozo et al., 2012), and if these compounds impact microbe‐microbe interactions.

Here, we use in vitro growth assays to test the degree to which nectar chemistry alone, or in combination with competitive dynamics, impacts microbial growth in a nectar analog. First, we tested the hypothesis that common nectar microbes can better tolerate a variety of nectar chemistries compared to microbes isolated from non‐nectar habitats. If non‐nectar specialists grow well in the presence of nectar compounds, it would indicate that filtering by these compounds is not a major driver of community assembly, and that other factors such as dispersal limitation or competition are more important. However, if only nectar specialists can maintain growth in the presence of common compounds found in nectar, it would suggest that environmental filtering may play a major role in nectar microbial community assembly. Second, we tested the hypothesis that the presence of nectar compounds affects the outcome of microbial competition in nectar.

## EXPERIMENTAL PROCEDURES AND RESULTS

### Microbial strains

We tested the effects of nectar compounds on the growth of the fungi *Metschnikowia reukaufii*, *Aureobasidium pullulans*, *Starmerella bombi*, *Rhodotorula fujisanensis*, *Saccharomyces cerevisiae*, *Zygosaccharomyces bailii*, and the bacteria, *Acinetobacter nectaris*, *Rosenbergiella nectarea*, *Bacillus subtilis*, *Pantoea agglomerans*, *Pseudomonas mandelii*, and *Pectobacterium carotovorum*. The species assayed include microbes commonly isolated from nectar, pollinators, and the environment (Table 1). We tested compounds detected in nectar that have been hypothesised or demonstrated to be antimicrobial and used concentrations in line with levels documented in nectar (Supplemental Table 1). We tested hydrogen peroxide (H_2_O_2)_, a reactive oxygen species found in some nectars, at two concentrations (2 and 4 mM, (Carter & Thornburg, 2004)); deltaline, a norditerpene alkaloid found in the nectar of *Delphinium spp*. and a potent toxin for eukaryotes (22 μg/ml, (Cook et al., 2013)); BrLTP2.1, a lipid transfer protein isolated from *Brassica rapa* nectar, hereafter referred to as LTP (150 μg/ml, (Schmitt et al., 2018)); linalool, a common volatile found in nectar (100 ng/ml, (Burdon et al., 2018)); ethanol (EtOH), a common byproduct of fermentation in nectar (1%, (Wiens et al., 2008)) and elevated sugar at 30%, along with a 15% base control nectar solution (which covers the low and moderate levels of natural sugar concentrations) (Nicolson et al., 2007). These compounds were chosen because they represent a broad range of compounds found across floral nectars and were feasible to obtain. See Supplemental Methods 2 for the recipes and process of creating control and treatment ‘nectars’.

**TABLE 1:** The microbes used in the study along with each strain's source

Microbe	Type	Family	Order	Class	Isolation source	Frequency of isolation in nectar
*Aureobasidium pullulans*	Ascomycete “black yeast”	*Dothioraceae*	*Dothioraceae*	*Dothioraceae*	*Epilobium canum* nectar	Medium [10]
*Metschnikowia reukaufii*	Ascomycete yeast	*Metschnikowiaceae*	*Saccharomycetales*	*Saccharomycetes*	*Epilobium canum* nectar	High [10, 27]
*Rhodotorula fujisanensis*	Basidiomycete yeast	*Sporidiobolaceae*	*Sporidiobolaceae*	*Microbotryomycetes*	*Ranunculus californicus* nectar	Medium [11]
*Saccharomyces cerevisiae*	Ascomycete yeast	*Saccharomycetaceae*	*Saccharomycetales*	*Saccharomycetes*	Unidentified flower	Low*
*Starmerella bombi*	Ascomycete yeast	*Incertae sedis*	*Saccharomycetales*	*Saccharomycetes*	*Bombus vosnesenskii* queen regurgitant	Medium [27]
*Zygosaccharomyces bailii*	Ascomycete yeast	*Saccharomycetaceae*	*Saccharomycetales*	*Saccharomycetes*	*Apis mellifera*	Low*
*Acinetobacter nectaris*	Bacteria	*Moraxellaceae*	*Pseudomonadales*	*Gammaproteobacteria*	*Penstemon heterophyllus* nectar	High [11, 32]
*Bacillus subtilis*	Bacteria	*Bacillaceae*	*Bacillales*	*Bacilli*	*Epilobium canum* nectar	Medium [11, 32]
*Pantoea agglomerans*	Bacteria	*Erwiniaceae*	*Enterobacterales*	*Gammaproteobacteria*	*Calystegia occidentalis* nectar	High [11, 32]
*Pectobacterium carotovorum*	Bacteria	*Pectobacteriaceae*	*Enterobacterales*	*Gammaproteobacteria*	*Solanum tuberosum*	Low [11]
*Pseudomonas mandelii*	Bacteria	*Pseudomonadaceae*	*Pseudomonadales*	*Gammaproteobacteria*	*Bombus vosnesenskii* queen regurgitant	Medium [11]
*Rosenbergiella nectarea*	Bacteria	*Enterobacteriaceae*	*Enterobacterales*	*Gammaproteobacteria*	*Epilobium canum* nectar	High [11, 32]

*Note*: The prevalence score is an approximation based on the frequency microbes have been discovered in nectar microbe surveys. The star indicates we are not aware of this species being documented as isolated from floral nectar.

### Plate reader growth assay

To test the effect of individual compounds on the growth of single microbe species, we used 96 well plate growth assays and synthetic nectars to observe the change in optical density (OD) as a proxy for microbial growth with OD measurements at 600 nm every 15 min for 72 h. We used mathematical models to fit logarithmic curves to OD measurements and adjusted wells to account for plate effects (see Supplemental figure 1 plate mapping). To compare a treatment's relative impact on growth across microbes, we computed a scaled value of growth rate (**μ**) and maximum growth (**A**) by adjusting each microbe's growth in treatment relative to their growth in control nectar across all plates [*log* ((*scaled value = treatment* **μ** or **A** /*mean control* **μ** or **A**) + *1*)]. A scaled value over *log*(*2*) indicates a treatment **μ** or **A** greater than that microbe's control and scaled value below *log*(*2*) indicates a **μ** or **A** lower than that microbe's control. These transformations allow us to compare the effects of nectar compounds across many microbes that varied in absolute growth. See supplemental Methods 3 for all data analysis.

### Treatment impacts across all microbes

**FIGURE 1:**
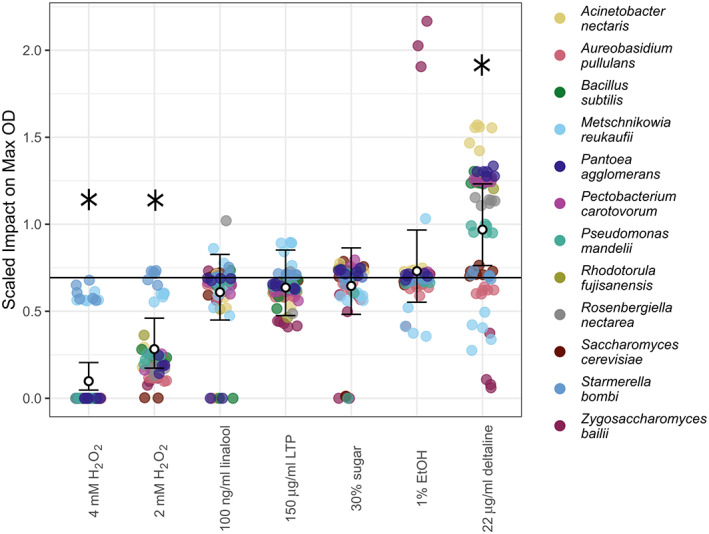
Nectar compounds differ in their effects on maximum microbial density. The *Y*‐axis indicates the scaled effect of treatment on maximum OD (optical density) compared to control nectar. A horizontal line is added at *Y* = log(2). Values above this line represent an increase in maximum density compared to controls and values lower indicate a decrease in maximum density. White points and bars show the negative binomial model coefficient and 95% confidence intervals for each compound. Coloured points indicate individual replicates for each microbe and contain a slight horizontal jitter to aid in readability. Stars represent significant overall treatment impacts at *p* < 0.05

Nectar compounds differed in their effect on maximum scaled OD (Figure 1); H_2_O_2_ strongly suppressed the growth of most microbes at 2 mM (negative binomial model coefficients and standard error: −0.9 ± 0.27, *p* < 0.001) and 4 mM (−1.95 ± 0.39, *p* < 0.001). 30% sucrose (−0.07 ± 0.18, *p* = 0.7), LTP (−0.08 ± 0.18, *p* = 0.64), linalool (−0.13 ± 0.18, *p* = 0.49) and EtOH had no significant effect (0.06 ± 0.17, *p* = 0.75). In contrast, the diterpene alkaloid deltaline increased maximum OD overall (0.34 ± 0.15, *p* = 0.03). Scaled maximum OD was correlated with scaled maximum growth rate (*r* = 0.67, *p* < 0.001) and effects of treatments on both were congruent, although not identical (Supplemental Figure 2).

### Microbe‐specific response to treatments

**FIGURE 2:**
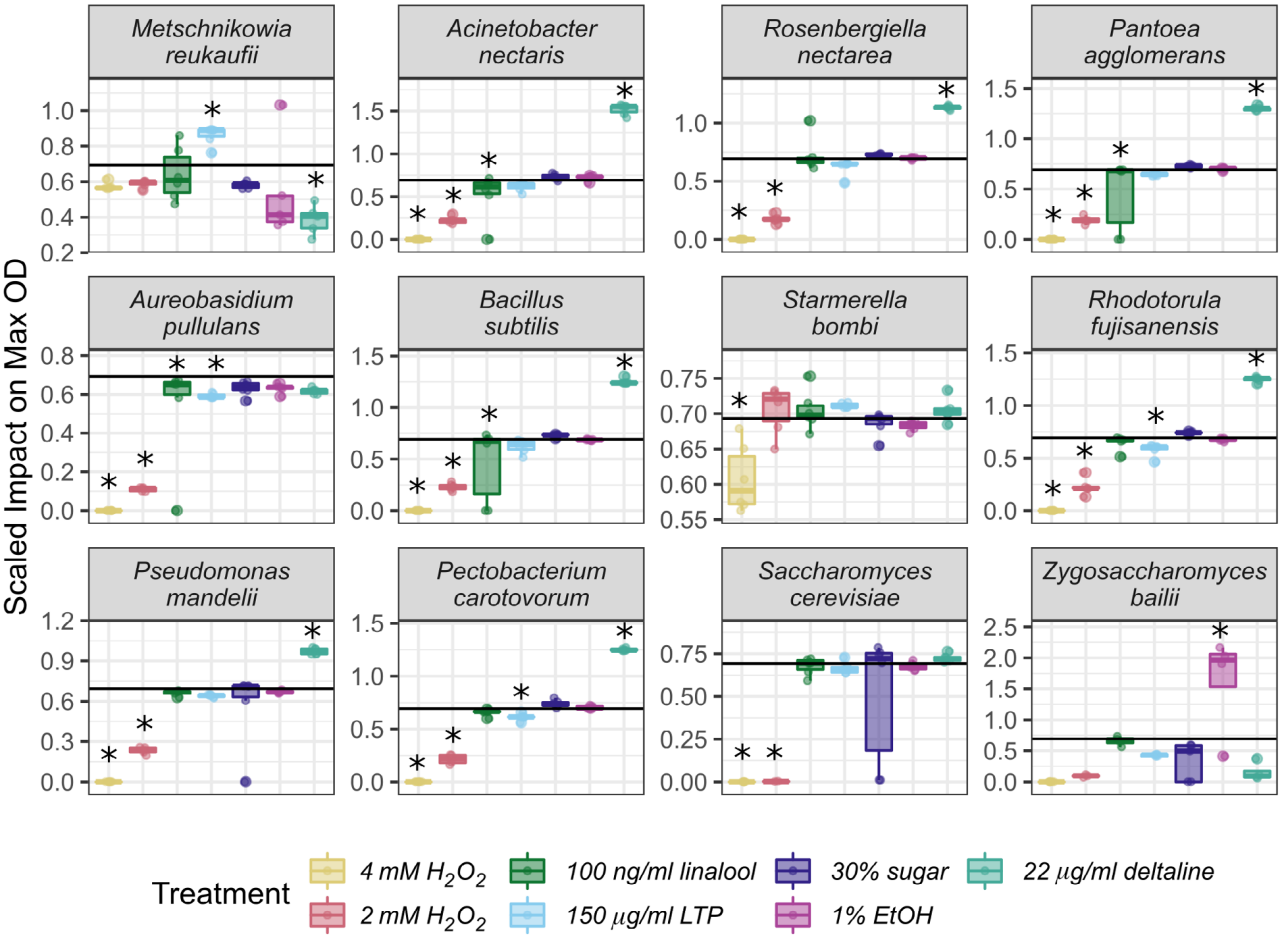
Microbial species vary in the scaled impact of treatment on maximum optical density. The *Y*‐axis is the scaled impact of a treatment on a microbe's maximum OD compared to controls, as in Figure 1, but separated to more clearly display variation among species. Microbes are ordered from most frequently (top left) to least frequently isolated from nectar (bottom right). Stars indicate significant treatment impact on maximum OD compared to control (*p* < 0.05). See Supplemental Figure 3 for non‐scaled data

**FIGURE 3:**
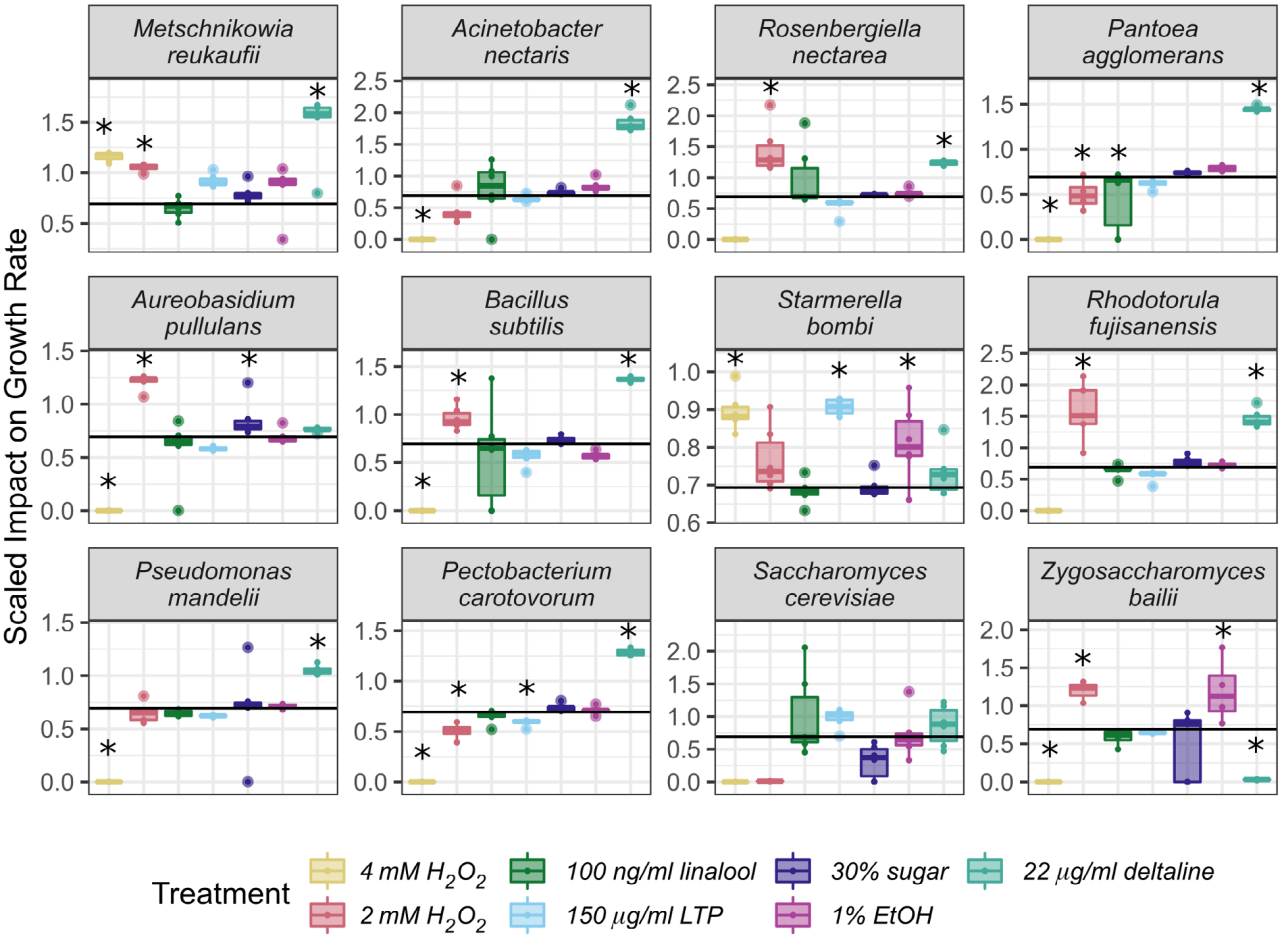
Microbial species vary in the scaled impact of treatment on growth rate. The *Y*‐axis is the scaled impact of a treatment on a microbe's growth rate compared to controls, as in Figure 1, but separated to more clearly display variation among species. Microbes are ordered from most frequently (top left) to least frequently isolated from nectar (bottom right). Stars indicate significant treatment impact on maximum OD compared to control (p < 0.05). See Supplemental Figure 4 for non‐scaled data

**FIGURE 4:**
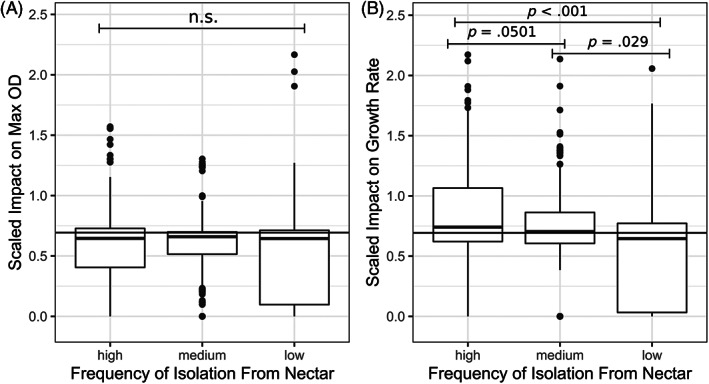
Microbial isolation source predicts sensitivity of growth rate but not maximum OD to treatments. The *Y*‐axis indicates the scaled effect of treatment on maximum OD (panel A) and growth rate (panel B) compared to control nectar. A horizontal line is added at *Y* = log(2). Values above this line represent an increase in maximum density compared to controls and values lower indicate a decrease in maximum density

Microbial species varied in their maximum OD and growth rate in control nectar and in response to treatment additions (Supplemental Figures 3 and 4, *p* < 0.05). All microbes were impacted by at least one treatment, but treatments differed in their effect on maximum OD (Figure 2) and growth rate (Figure 3) across microbial species. Species' responses to nectar composition depended on the specific nectar compound tested: no microbe had significantly reduced maximum OD or growth rate across all treatments (Figure 2). When comparing across all treatments, the scaled maximum OD was not significantly different across degrees of nectar specialisation (*p* > 0.05; Figure 4A), however, scaled growth rate was significantly different: microbes infrequently isolated from nectar had a lower scaled growth rate than both the highly and medium specialised group (*p* < 0.05; Figure 4B).

### Differences between yeast and bacteria

Yeasts and bacteria differed significantly in the maximum OD attained, with yeasts (0.82 ± 0.35, *p* = 0.04) having a higher max OD than bacteria (0.01 ± 0.25, *p* = 0.96) (Supplemental Figure 5A). When assaying treatments' scaled impact on max OD, yeast (−0.05 ± 0.12, *p* = 0.67) were significantly less affected by treatments compared to bacteria (−0.7 ± 0.25, *p* = 0.004) (Supplemental Figure 5B), suggesting that yeasts may be more resistant to the inhibitory effects of nectar chemicals than bacteria. However, there was no significant phylogenetic signal present that was driving the scaled max OD (*λ* = 0.59, *p* = 1; *K* = 0.2, and *p* = 0.81) or growth rate (*λ* = 0.2, *p* = 1; *K* = 0.19, and *p* = 0.91) indicating that while bacteria and yeasts as a whole may broadly differ, there is strong variation within each kingdom and relatedness does not drive the response to nectar chemistry (Supplemental Figure 6).

### Co‐growth experiment

To test if nectar composition could shift microbial interactions, we grew pairs of microbes across several treatment solutions: (1) *S. bombi & Z. bailii* (a facultative nectar yeast with a non‐nectar yeast), (2) *M. reukaufii & R. nectarea* (a nectar specialist yeast with a nectar specialist bacteria), and (3) *S. cerevisiae & R. nectarea* (a non‐nectar specialist yeast with a nectar specialist bacteria). We also ran a pairing of *M. reukaufii & S. cerevisiae*, however, the vial lids burst open during incubation due to extremely rapid fermentation. These species pairings were chosen from many cogrowth combinations as they produced colonies that were easily distinguishable from one another during preliminary cogrowth tests. If the dominance of nectar specialists is driven by nectar chemicals shifting microbe‐microbe competition we predict nectar specialists will increase in relative abundance in the presence of nectar compounds, while the relative performance of environmental microbes should be reduced compared to control co‐growth trials. We chose a subset of treatments for co‐growth assays, including 4 mM H_2_O_2_, 22 μg/ml deltaline, 100 ng/ml linalool, and 1% EtOH. Treatments used the same recipes as the growth experiments described above. See supplemental Methods 3 for full experimental procedure.

**FIGURE 5:**
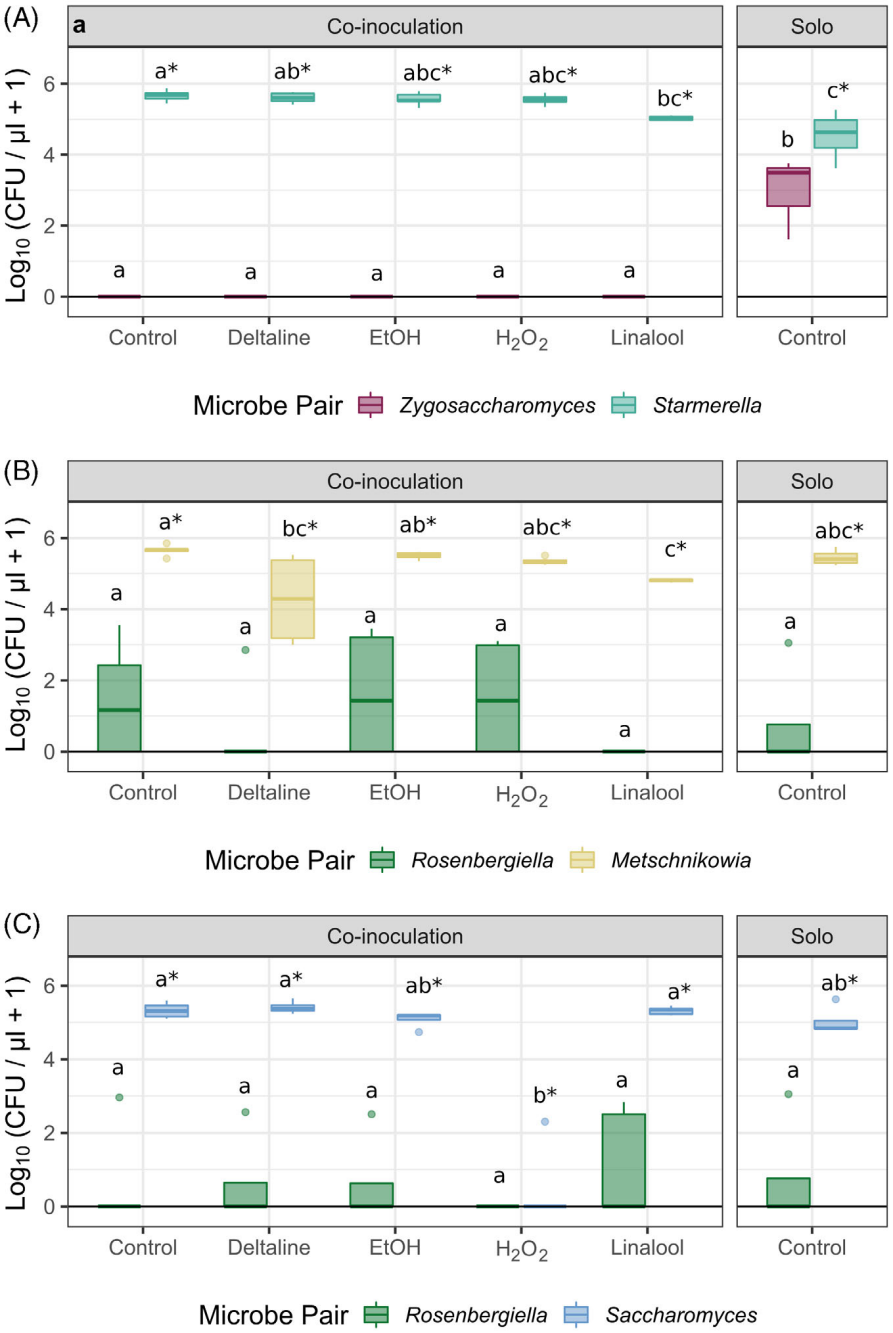
Nectar compounds influence microbial community outcomes but differ depending on species considered. The colony forming units (CFUs) per μl of synthetic nectar formed by microbes grown in co‐culture and alone across different nectar chemistries. Each panel represents a different pairing of microbes; panel A pairs a facultative nectar yeast with a non‐nectar yeast (*Starmerella bombi* and *Zygosaccharomyces bailii*), panel B pairs a nectar specialist yeast with a nectar specialist bacteria (*Metschnikowia reukaufii* and *Rosenbergiella nectarea*), and panel C pairs a non‐nectar specialist yeast with a nectar specialist bacteria (*Saccharomyces cerevisiae* and *Rosenbergiella nectarea*). Letters indicate significant pairwise differences between treatments (*p* < 0.05) and are shown separately for each microbe

The presence of competitors and nectar compounds together affected microbial abundance after 3 days for all species pairings (Figure 5). For example, in a co‐culture of the food spoilage specialist *Z. bailii* and bee‐associated *S. bombi*, *Z. bailii* never formed CFUs in the presence of a competitor, but did when grown alone, suggesting strong competitive exclusion. In contrast, *S. bombi* in the same pairing showed increased CFU formation in co‐culture relative to its growth alone (*p* < 0.001), in control nectar, 22 μg/ml deltaline, 1% EtOH, and 4 mM H_2_O_2_ treatment nectars (Figure 5A). In the pairing of two nectar ‘specialists’, neither the bacteria *R. nectarea* nor the yeast *M. reukaufii* showed an altered CFU density in co‐culture compared to growth in isolation (Figure 5B). When co‐culturing *R. nectarea* and *S. cerevisiae*, we found that contrary to our original hypothesis, the non‐nectar yeast *S. cerevisiae* did not show a significant reduction (*p* > 0.05) in growth compared with growth alone. Notably, however, the addition of H_2_O_2_ reduced *S. cerevisiae* and made *R. nectarea* growth undetectable (Figure 5C)*—*in contrast to the ability of *R. nectarea* to persist in the presence of *M. reukaufii* in H_2_O_2_‐containing nectar.

## DISCUSSION

All nectar constituents tested had species‐specific effects on microbial growth, significantly impacting certain microbes while showing no impact on others. Hydrogen peroxide showed strong antimicrobial properties across most microbes assayed, both nectar specialists and non‐specialists. It is unknown how common H_2_O_2_ is in nectar, but it has been detected in several genera of plants including *Nicotiana* and *Cucurbita* (Carter et al., 2007; Nocentini et al., 2015). Despite strong suppressive effects on most species (including those with documented catalase activity) (Álvarez‐Pérez et al., 2012), the antimicrobial effect of H_2_O_2_ was not universal. Notably the maximum OD of the yeast *M. reukaufii* and *Z. bailli* were unaffected by any concentration of H_2_O_2_ tested and *S. bombi* was only affected at 4 mM. It should be noted, however, that H_2_O_2_ has a very short half‐life and likely degraded over the course of each assay. In floral nectar, H_2_O_2_ can be continuously produced suggesting that our study may underestimate its antimicrobial properties. Other tested compounds were more selective in their growth suppression and impacted different microbes including those frequently and seldom isolated from nectar. We only tested 1 isolate per species here, but it is possible there could be strain specific adaptation or susceptibility to different compounds. This is an intriguing hypothesis for future work.

The observed differences in the selectivity of compounds suggest that nectar antimicrobial compounds (NACs) may fall into two broad classes with different functions: general antimicrobials and selective filters. General NACs (e.g., H_2_O_2_ here) may keep a flower from being colonised by most microbes and are possibly common in nature. In some ecosystems as many as 80% of plants have no culturable yeasts and some have very low incidence of culturable bacteria (Herrera et al., 2009; Vannette et al., 2021). We predict that general NACs, or other mechanisms to limit microbial growth, might be more common in ecosystems where plants have a high likelihood of colonisation by antagonistic microbes but a low probability of colonisation by beneficial microbes (or where the costs of antagonists consistently outweigh the benefits of mutualists). Conversely, we predict that selective filtering NACs might be more common in ecosystems where plants have equal likelihoods of being colonised by beneficial or antagonistic microbes. Direct effects of NACs on pollinator behaviour and health, however, should not be discounted and likely also plays a role in the selection on NACs (Manson et al., 2013). While we lack data on the plant traits that shape communities of antagonistic and beneficial microbes (Adler et al., 2021), and there are likely other modes beyond NACs that work in conjunction such as floral morphology or other nectar constituents including enzymes, ions, lipids, among others, these data suggest that selective NACs may be one route by which plants shape their nectar microbiome. However, with the extreme diversity in floral nectar chemistry, many general and selective NACs have likely not yet been identified or may escape notice by being context dependent. Characterising the relative abundance of general and selective NACs across different microbial landscapes might be particularly fruitful in disentangling how microbes shape selection on nectar traits.

Our findings suggest that NACs can also shift competitive dynamics and the trajectories of nectar microbial communities as previously suggested (Álvarez‐Pérez et al., 2019). While we found no relationship between degree of nectar specialization and treatment impacts on maximum growth, the growth rate of non‐nectar specialists was more suppressed in the presence of nectar compounds, and bacteria were more negatively affected than yeasts, both of which could affect end community assembly. Our co‐culture experiment further shows that treatments can impact communities not only by decreasing the growth of some microbes, but also increasing the growth of others in co‐culture. Here, *Z. bailii* did not grow in co‐cultures with *S. bombi*, however, *S. bombi* showed elevated growth in co‐culture, even in the presence of H_2_O_2_. We hypothesise that the presence of *Z. bailii* may have facilitated the growth of *S. bombi* by potentially providing additional nutrition. Alternatively, it appears that some microbes may facilitate each other's growth. For example, *R. nectarea* grew in H_2_O_2_‐containing nectar in the presence of *M. reukaufii* but not *S. cerevisiae*, perhaps suggesting that *M. reukaufii*, which itself does not appear to be impacted by H_2_O_2_, may have methods for detoxifying H_2_O_2_ that extend to other inhabitants of the same nectar environment.

The impact of plant chemistry on ecological interactions can be difficult to predict and some presumptive NACs may even benefit certain microbes. We predicted that the norditerpene alkaloid deltaline would broadly suppress microbial growth, but our results generally suggest otherwise. Deltaline only decreased the growth of *M. reukaufii*, with most other microbes increasing in maximum OD relative to their control. This is surprising considering that other norditerpene alkaloids, extracted from flowering plants in the same family as *Delphinium*, have strong antimicrobial properties (Ahmad et al., 2008). Prior work looking at the antimicrobial effects of norditerpenes, however, tested concentrations higher than those occurring in nectar (Ahmad et al., 2008). For microbes that do not experience growth suppression, it is possible that deltaline is a source of otherwise limiting compound such as nitrogen (Vannette & Fukami, 2014), although our study had much higher levels of nitrogen compared to most floral nectar (Nicolson et al., 2007). It is possible that compounds that might be otherwise anti‐microbial in growth media or in other plant tissues may benefit microbes in nectar. These findings highlight that generalising across plant tissues and among whole classes, or even subclasses, of compounds should be done with caution.

Although the impact of nectar secondary metabolites on microbes may be an understudied ecological role, other abiotic and biotic ecological drivers should also be considered. Nectar chemicals are widespread (Adler, 2000) but may be non‐adaptive consequences of chemical defence in other plant tissues (Adler, 2000; Adler et al., 2012) where they can effect florivores or pollinators and their behaviour (Wright et al., 2013). Additionally, nectar chemicals are often in low concentrations when compared to compounds in other plant tissues (Palmer‐Young et al., 2019). Compounds in other plant tissues may also influence the nectar environment and shape microbial communities, for instance, when pollen gets deposited into floral nectar. Nectar is a complex and dynamic solution, changing with enzyme activity, host‐mediated secretion and resorption, and via contact with floral tissues—all precluded by our use of synthetic nectar. It is possible that these complex interactions of chemicals may increase or decrease the effect of the specific compounds tested here. Whether the impacts of NACs observed here are stronger or weaker than these other factors (and thus are ecologically relevant) is an open question.

Taken together, our results suggest variable effects of nectar chemistry and that different microbes may be excluded from nectar for varying reasons. The findings that nectar compounds can shift microbial colonization and community dynamics raise more questions for further study. Given that nectar is chemically diverse (Palmer‐Young et al., 2019), and microbes vary in dispersal limitation (Vannette et al., 2021), what does the observed selectivity of NACs mean at a landscape scale? On one hand, it could lead to a diversity of microbial niches where different floral species have different selective NACs, and thus floral diversity would likely increase microbial diversity at the landscape scale. However, this is not found in nectar surveys, suggesting that other strong drivers, such as dispersal (Russell et al., 2019; Vannette et al., 2021), competitive ability (Dhami et al., 2016; Fukami, 2015), or intraspecific variation in microbial sensitivity to NACs, also contribute to low species diversity in floral microbial communities (Dhami et al., 2018; Herrera et al., 2014). Finally, given our result that nectar secondary chemistry can affect microbial growth, and may affect yeasts to a lesser extent than bacteria, characterising variation in antimicrobial potential among plant populations and species may allow a better understanding of how microbes, pollinators and other forces shape the ecology and evolution of nectar traits.

## AUTHOR CONTRIBUTIONS

Tobias G. Mueller and Rachel L. Vannette conceived of and designed the study with input provided by Jacob S. Francis. Data collection was performed by Tobias G. Mueller with help from Jacob S. Francis. Data analysis was performed by Tobias G. Mueller. The first draft of the manuscript was written by Tobias G. Mueller with all authors contributing to the writing and editing process. All authors read and approved of the final manuscript.

## FUNDING INFORMATION

This work was supported by the National Science Foundation (DEB‐1846266 to RLV) and the United States Department of Agriculture/Cooperative State Research, Education and Extension Service (Multistate NE1501 to RLV).

## CONFLICT OF INTEREST

The authors have no relevant financial or non‐financial interests to disclose.

## Supporting information


**Data S1:** supporting Information.

## Data Availability

All datasets generated during the study as well as data analysis scripts and outputs can be found on GitHub at https://github.com/tobiasgmueller/nectar_growth_assay.
