# Supplementary material for: Nectar compounds impact bacterial and fungal growth and shift community dynamics in a nectar analog
Source: Environ Microbiol Rep. 2023 Feb 13;15(3):170–80. doi: 10.1111/1758-2229.13139 (PMC10464699; doi:10.1111/1758-2229.13139)
Supplement: Supplementary file 1 — Data S1: supporting Information. [file EMI4-15-170-s001.docx]

**Title: Nectar compounds impact bacterial and fungal growth and shift community dynamics in a nectar analog**

**Authors**: Tobias G. Mueller^[1][2]^*, Jacob S. Francis^[1]^, Rachel L. Vannette^[1]^

[1] Department of Entomology and Nematology, University of California, Davis, Davis, CA, USA

[2] Department of Entomology, Cornell University, Ithaca, NY, USA

* Corresponding author; email: tm524@cornell.edu, (+1) 512-992-7688, 4124 Comstock Hall, Cornell University, Ithaca, NY, 14850

**Supplemental method 1.** The media recipes for the yeast media (YM) and tryptone soy agar (TSA) that fungi and bacteria were cultured on respectively

Yeast Media (YM)

To make 1000ml of YM, dissolve the following in deionized H_2_O

- 3g Malt Extract
- 5g Peptone
- 10g Glucose (Dextrose)
- 20g Agar
- 3g Yeast Extract

after autoclaving add

- 1mL Chloramphenicol (100 mg/mL)

Tryptone Soy Agar Media (TSA)

To make 1000ml of TSA, dissolve the following in deionized H_2_O

- 15g Tryptone
- 15g Agar
- 5g Soytone
- 5g NaCl
- 50g Fructose

after autoclaving add

- 1mL Cycloheximide (100mg/mL)

**Supplemental Methods 2.**

Creating Microbial suspensions

To standardize microbial density across treatments, we created master microbial suspensions. First, we grew microbial stocks for three days on Yeast Media Agar (YMA) containing chloramphenicol (at 100 mg/L to reduce bacterial growth) or fructose enhanced Trypticase Soy Agar (TSA) containing cycloheximide (at 100 mg/L to reduce fungal growth) for fungi and bacteria respectively (Supplemental Method 1). We then created 2000 cell/μL suspensions (quantified via hemocytometer) in 15% glycerol v/v with 15% sucrose w/v and stored aliquots at -80^o^C for the duration of the experiment. Each plate or cogrowth assay used a new aliquot to ensure that every replicate had the same starting cell densities of each focal microbe. We verified that there was no decrease in microbial viability from length of freezing by correlating the lag time of microbes grown in control nectar to length of time frozen (r = -.13, p = .08).

*Preparing synthetic nectars*

To prepare the synthetic nectars (treatment and control solutions), we weighed dry reagents on a microbalance to a precision of 0.0025 grams before washing them into a volumetric flask and dissolving the reagents in DI water. Liquid reagents were added and then the entire solution was diluted with DI water to the proper concentration before being vortexed and sterilized using a syringe filter (0.2 μm cellulose acetate membrane, Corning, Corning NY, product number 431219). Base nectar consisted of 15% sugar (50:25:25 sucrose:glucose:fructose) w/v, 1% peptone w/v, 3% yeast extract w/v, 50% 100x non-essential amino acids v/v. This high nitrogen nectar analog was chosen so that all tested microbes could grow in the base solution to detectable levels, allowing us to test the impacts of nectar compounds alone on growth separately from the effects of nutrient limitation, however, it is possible that nutrient limitation and growth suppressive compounds may work in conjunction in floral nectar.

**Supplemental methods 3.**

*Data Analysis*

All analyses were performed in RStudio using R version 4.0.1 (RStudio Team, 2020).

*Curve fitting and data curation*

We used the Grofit package (Kahm *et al.*, 2010) to fit logarithmic curves to the optical density (OD) timeseries. The initial OD value for each well was deducted from all readings to account for starting solution OD. Best-fit growth curve models were selected using AIC and each fitted curve was visually inspected after which growth rate (𝛍) and maximum OD (𝚨) were extracted from the fitted curves.

To ensure data quality, we performed the following checks on all growth curves before curve fitting: if a well did not change OD over 72 hours, the 𝚨 and 𝛍 were set to zero. If the OD increased and then returned to the starting value within 72 hours, we considered the well having no growth and set both parameters to zero. This was likely driven by a period of growth followed by cell death and in turn decreased OD. While it is possible that clumping of cells could lead to a slight decrease of OD, plates showing this growth pattern were visually inspected under a microscope after 72 hours and no evidence of clumping in wells was found. If only 1 treatment well out of the 6 did not grow, we considered this to be due to an error (possibly no microbe addition) and removed the well from the analysis. If no mathematical fit could be plotted to the OD readings, we removed the well from the analysis (less than 5% of growth curves). Many of these unfittable wells showed flatline curves with a single value change during the 72 hours likely not caused by microbial growth. One well showed an 𝚨 100 times greater than all other wells in that treatment and was removed. For a single plate (deltaline), condensation caused a temporary drop in the OD for the first 45 minutes. For these wells we set the starting OD as the lowest OD reading from the first hour.

To control for variation in growth among plates, we divided the mean growth of control wells for each microbe on each plate by the mean growth of that microbe’s control wells across all plates giving us a plate-specific growth ratio. We then multiplied the treatment wells on a given plate by that plate-specific ratio.

*Treatment impacts across all microbes*

To compare the effects of treatment across all microbes we fit a negative binomial model (Venables *et al.*, 2002) with scaled maximum OD as a function of treatment. To test if the scaled maximum OD and scaled growth rate were correlated we calculated the Pearson’s correlation coefficient.

*Microbe-specific response to treatments*

We compared each microbe’s growth in different nectar chemistries to their growth in control nectar using a Kruskal-Wallis test followed by a Dunnett’s test (Signorell, 2021), with separate models for maximum OD and maximum growth rate. To test if treatment impacts were related to the frequency microbes occur in nectar (hereafter referred to as nectar specialization) we ranked microbes as “high”, “medium”, or “low” according to their relative incidence and abundance in nectar (Table 1). We ran a Kruskal-Wallis test comparing scaled maximum OD and scaled growth rate to the level of specialization. Significant results were followed by a Dunn’s test with a Holm-Bonferroni correction to compute pairwise differences (Signorell, 2021).

*Differences between yeast and bacteria*

To compare if nectar chemistry differentially affected yeasts compared to bacteria, we used a linear random effects model (for maximum OD) and negative binomial model (for scaled maximum OD) comparing microbial growth between kingdoms, with microbe and treatment as random intercept terms.

*Co-growth assay*

To compare how nectar chemistry can change community dynamics, we used a Kruskal-Wallis test followed by a Dunn’s test comparing each microbe's growth in co-culture across different nectar chemistries and alone in control nectar.

*Phylogenetic signal*

To determine if there was an effect of relatedness on the growth or inhibition of microbes, we calculated both Pagel’s lambda as well as Blomberg’s K using the phytools package in R. We tested for phylogenetic signals in sclaed maximum growth as well as scaled growth rate. Phylogenetic trees with divergence times were constructed using TreeTime of Life (Kumar *et al.*, 2022). Since divergence data wasn’t available for all species, *Rhodotorula fujinensis*, was replaced with *Rhodotorula graminis* and *Auerobasidium pullulans* was replaced with the co-family member *Sydowia polyspora*, however, neither substitution will impact divergence time in relation to other members of the tree.

**References**

Kahm, M., Hasenbrink, G., Lichtenberg-Fraté, H., Ludwig, J., and Kschischo, M. (2010) grofit: Fitting Biological Growth Curves with R. *Journal of Statistical Software* **33**: 1–21.

Kumar, S., Suleski, M., Craig, J.M., Kasprowicz, A.E., Sanderford, M., Li, M., et al. (2022) TimeTree 5: An Expanded Resource for Species Divergence Times. *Molecular Biology and Evolution* **39**: msac174.

RStudio Team (2020) RStudio: Integrated Development for R.

Signorell, A. (2021) DescTools: Tools for descriptive statistics.

Venables, W.N., Ripley, B.D., and Venables, W.N. (2002) Modern applied statistics with S, 4th ed. New York: Springer.

**Supplemental methods 4.**

*Co-growth experiment*

We performed the co-growth experiment in 200μL 8-strip PCR tubes with 6 tubes per treatment–microbe combination. Each tube consisted of 190μL of synthetic nectar and 5μL (10,000 cells) of each microbial freezer stock in that pairing. We vortexed the tubes for 5 seconds and incubated them at 25°C for 72 hours. To assess the effect of co-growth, we also grew each microbe in isolation, following the same methods, with tubes consisting of 190μL of synthetic nectar and 5μL of microbial suspension (N=4).

After 72 hours of incubation, we serially diluted the microbial suspensions and plated 100μL of diluted microbial suspension onto TSA and YMA plates. We diluted TSA plates 2x (plating 50μL of original suspension), YMA plates from the *Starmerella bombi* and *Zygosaccharomyces* pairing both 20x (5μL plated) and 200x (0.5μL plated), and YMA plates with *Metschnikowia* or *Saccharomyces* 200x (0.5μL plated). We chose these dilutions as they created countable CFUs. We then incubated plates at 25°C for 72 hours to allow microbial colonies to form, after which we counted the number of colonies per plate. *Rosenbergiella* did not form single colonies and instead the percent of the plate covered by growth was estimated and adjusted relative to the maximum CFU count of its yeast pairing (100% coverage = maximum CFU count).

| **Treatment** | **Levels Found in Nectar** | **Citations** |
| --- | --- | --- |
| **4 mM H_2_O_2_** | H_2_O_2_ levels up to 4mM have been found in ornamental tobacco (*Nicotiana langsdorffii × Nicotiana sanderae*) nectar | Carter, C. et al. Tobacco Nectaries Express a Novel NADPH Oxidase Implicated in the Defense of Floral Reproductive Tissues against Microorganisms. Plant Physiol 143, 389–399 (2007); Carter, C. & Thornburg, R. W. Is the nectar redox cycle a floral defense against microbial attack? Trends in Plant Science 9, 320–324 (2004) |
| **2 mM H_2_O_2_** |  |  |
| **30% Sugar** | Sugar levels in nectar can range from 8% to over 80% | Baker, H. G. Sugar Concentrations in Nectars from Hummingbird Flowers. Biotropica 7, 37–41 (1975); Herrera, C. M., Canto, A., Pozo, M. I. & Bazaga, P. Inhospitable sweetness: nectar filtering of pollinator-borne inocula leads to impoverished, phylogenetically clustered yeast communities. Proceedings of the Royal Society B: Biological Sciences 277, 747–754 (2010) |
| **100 ng/ml Linalool** | Linalool levels can range from 5ng to over 100ng/ml in *Penstemon digitalis* nectar | Burdon, R. C. F., Junker, R. R., Scofield, D. G. & Parachnowitsch, A. L. Bacteria colonising Penstemon digitalis show volatile and tissue-specific responses to a natural concentration range of the floral volatile linalool. Chemoecology 28, 11–19 (2018) |
| **150 μg/ml BrLTP2.1 (LTP)** | Exact concentrations are unknown, however, fluorescence of BrLTP2.1 shows high levels in *brassica rapa* nectar. Previous experiments tested up to 300μg/ml | Schmitt, A. J. et al. The major nectar protein of Brassica rapa is a non-specific lipid transfer protein, BrLTP2.1, with strong antifungal activity. J Exp Bot 69, 5587–5597 (2018) |
| **22 μg/ml Deltaline** | Deltaline levels can be up to .63μg/100mg in *Delphinium* nectar, however, concentrations of the norditerpene alkaloid class as a whole can reach up to 22μg/ml in *Delphinium* nectar | Cook, D., Manson, J. S., Gardner, D. R., Welch, K. D. & Irwin, R. E. Norditerpene alkaloid concentrations in tissues and floral rewards of larkspurs and impacts on pollinators. Biochemical Systematics and Ecology 48, 123–131 (2013) |
| **1% Ethanol** | The highest reported level of ethanol in nectar is 3.8%, however, no formal survey of ethanol in floral nectar has been performed | Wiens, F. et al. Chronic intake of fermented floral nectar by wild treeshrews. PNAS 105, 10426–10431 (2008) |

**Supplemental Table 1** The concentrations of nectar compounds used as treatments along with their reported natural concentrations in floral nectar

| **Treatment** | Base nectar | 30% sugar | 2mM H_2_O_2_ | 4mM H_2_O_2_ | 100 ng/mL Linalool | 150 μg/mL LTP | .22 μg/mL Delaline | 1% Ethanol |
| --- | --- | --- | --- | --- | --- | --- | --- | --- |
| **Total made** | **25 mLs** | **25 mLs** | **25 mLs** | **25 mLs** | **25 mLs** | **15mL** | **25 mLs** | **25 mLs** |
| **g Sucrose** | 1.875 | 3.75 | 1.875 | 1.875 | 1.875 | 1.125 | 1.875 | 1.875 |
| **g Glucose** | 0.9375 | 1.875 | 0.9375 | 0.9375 | 0.9375 | 0.5625 | 0.9375 | 0.9375 |
| **g Fructose** | 0.9375 | 1.875 | 0.9375 | 0.9375 | 0.9375 | 0.5625 | 0.9375 | 0.9375 |
| **g Peptone** | 0.25 | 0.25 | 0.25 | 0.25 | 0.25 | 0.15 | 0.25 | 0.25 |
| **g Yeast Extract** | 0.75 | 0.75 | 0.75 | 0.75 | 0.75 | 0.45 | 0.75 | 0.75 |
| **mL 100x non-essential amino acids** | 12.5 | 12.5 | 12.5 | 12.5 | 12.5 | 7.5 | 12.5 | 12.5 |
| **μL 30% H2O2** | - | - | 5.67 | 11.33 | - | - | - | - |
| **μL linalool** | - | - | - | - | 2.87 | - | - | - |
| **μg LTP** | - | - | - | - | - | 2250 | - | - |
| **mg Deltaline** | - | - | - | - | - | - | 0.55 | - |
| **μL 100% Ethanol** | - | - | - | - | - | - | - | 250 |

**Supplemental Table 2** Recipes for synthetic nectar treatment solutions. All treatments were fully dissolved in deionized water before being syringe filtered through a .2μm filter to ensure sterility


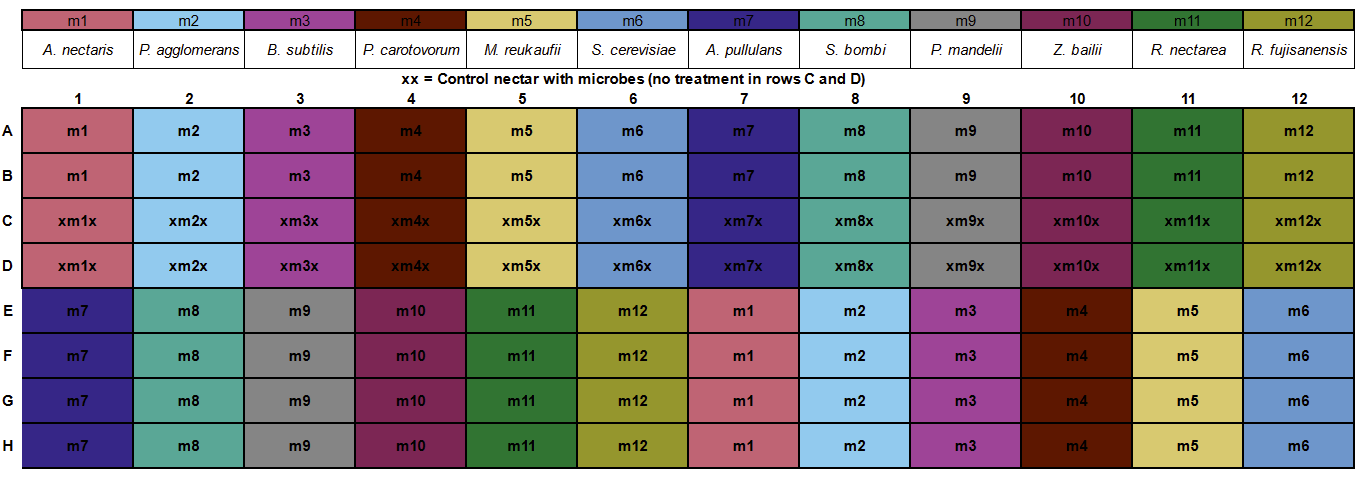


**Supplemental Figure 1** The layout of microbes on the 96 well plate. Each microbe (m1-m12, listed above) had 6 replicates in each treatment nectar (rows A:B, and E:H) and 2 replicates in control nectar (rows C:D) marked above with an X. The placement of each microbe on the plate was determined with a random number generator and kept consistent across all assays


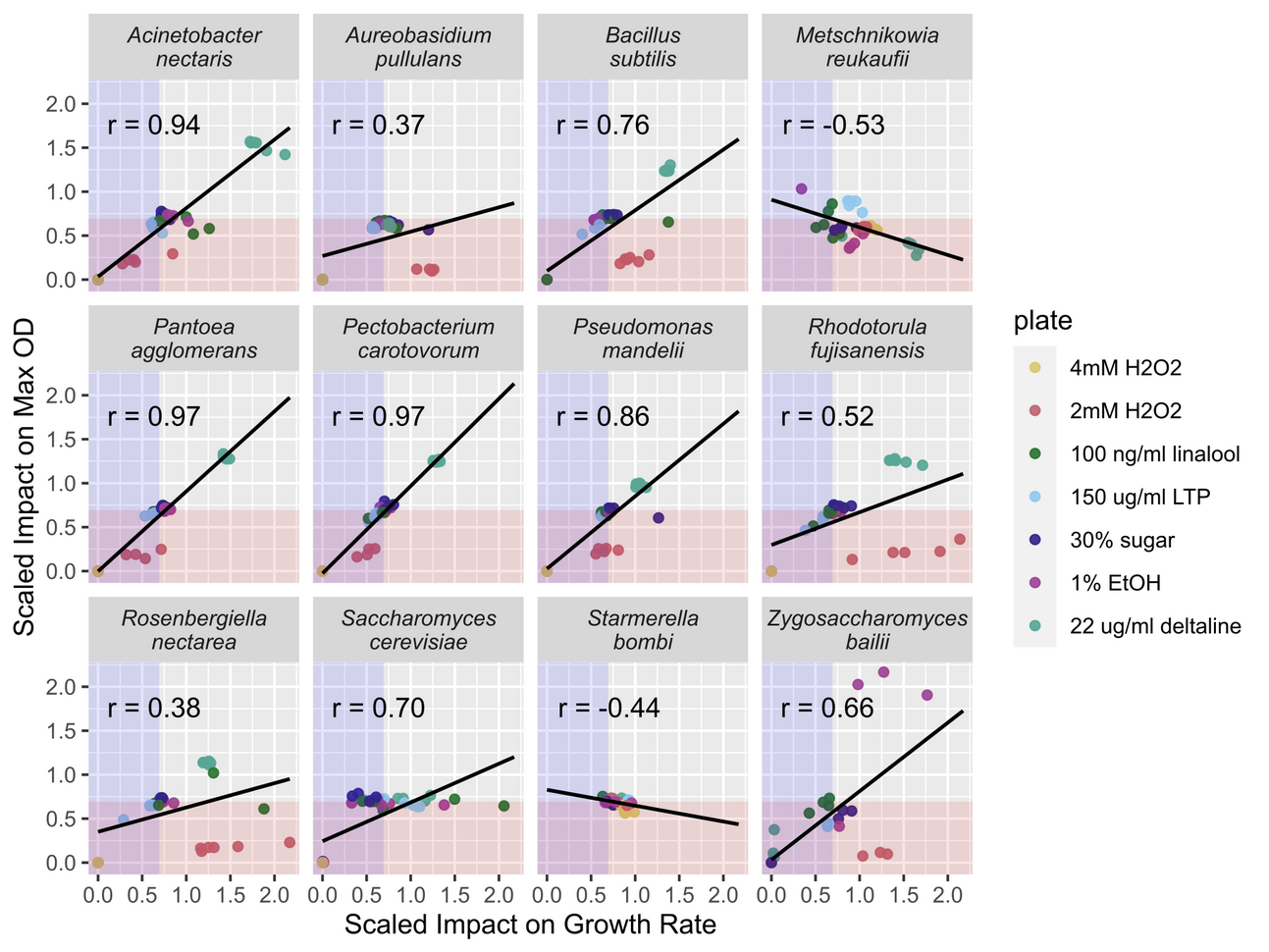


**Supplemental Figure 2** The treatment impacts on maximum OD and growth rate were correlated across many but not all species. The axes indicate the scaled effect of treatment compared to control nectar. Points inside the red/blue shaded area had a lower OD/growth rate, in treatment than control solutions. Points in both the blue and red shaded area had both a lower OD *and* growth rate in treatment than control solutions. The Pearson's correlation coefficient (r) is given for each microbse


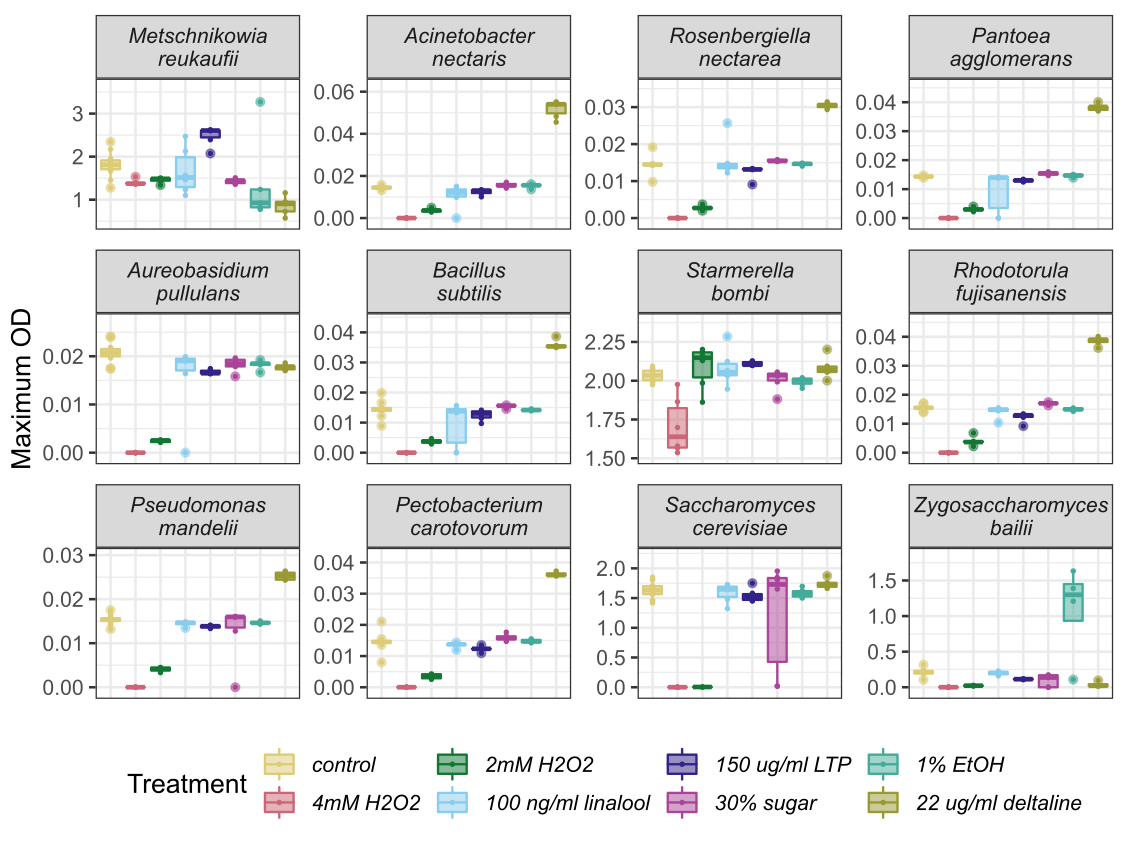


**Supplemental Figure 3.** Microbes differed in their maximum OD across different treatment nectars. Microbes are ordered from most frequently (top left) to least frequently isolated from nectar (bottom right)


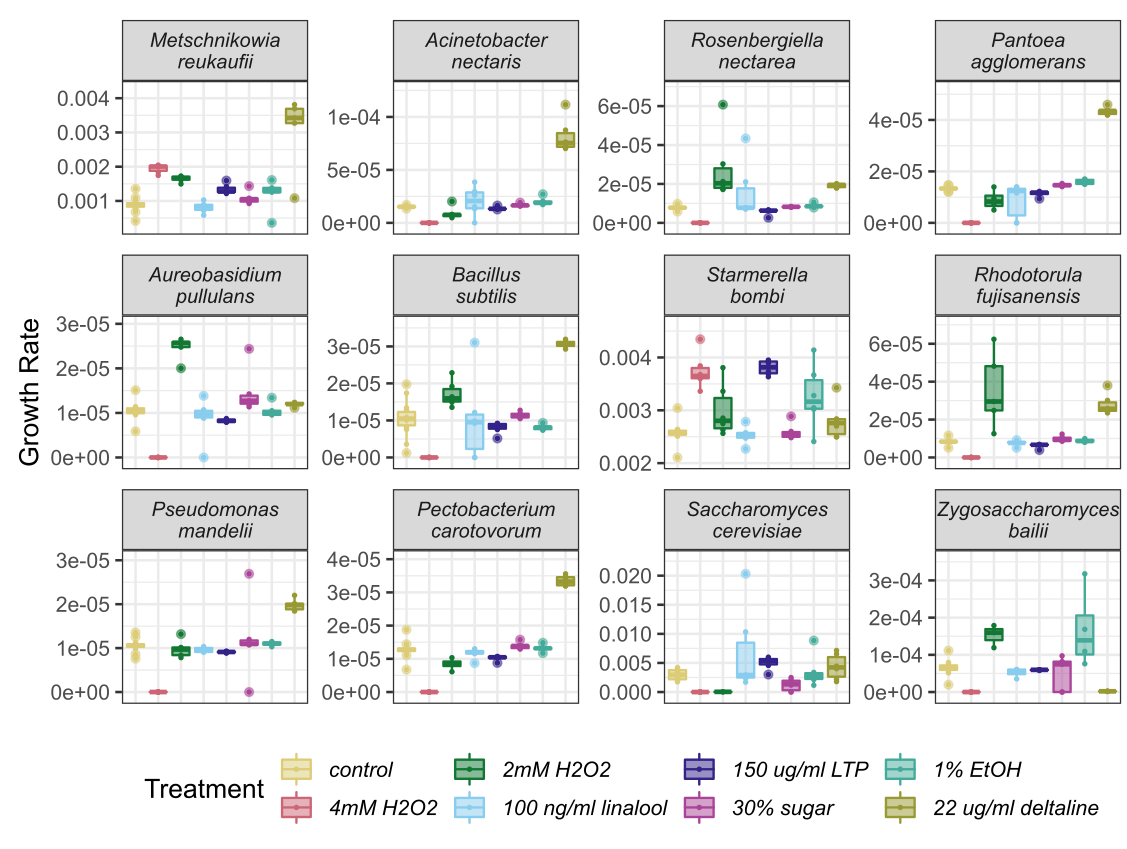


**Supplemental Figure 4.**  Microbes differed in their growth rate across different treatment nectars. Microbes are ordered from most frequently (top left) to least frequently isolated from nectar (bottom right)


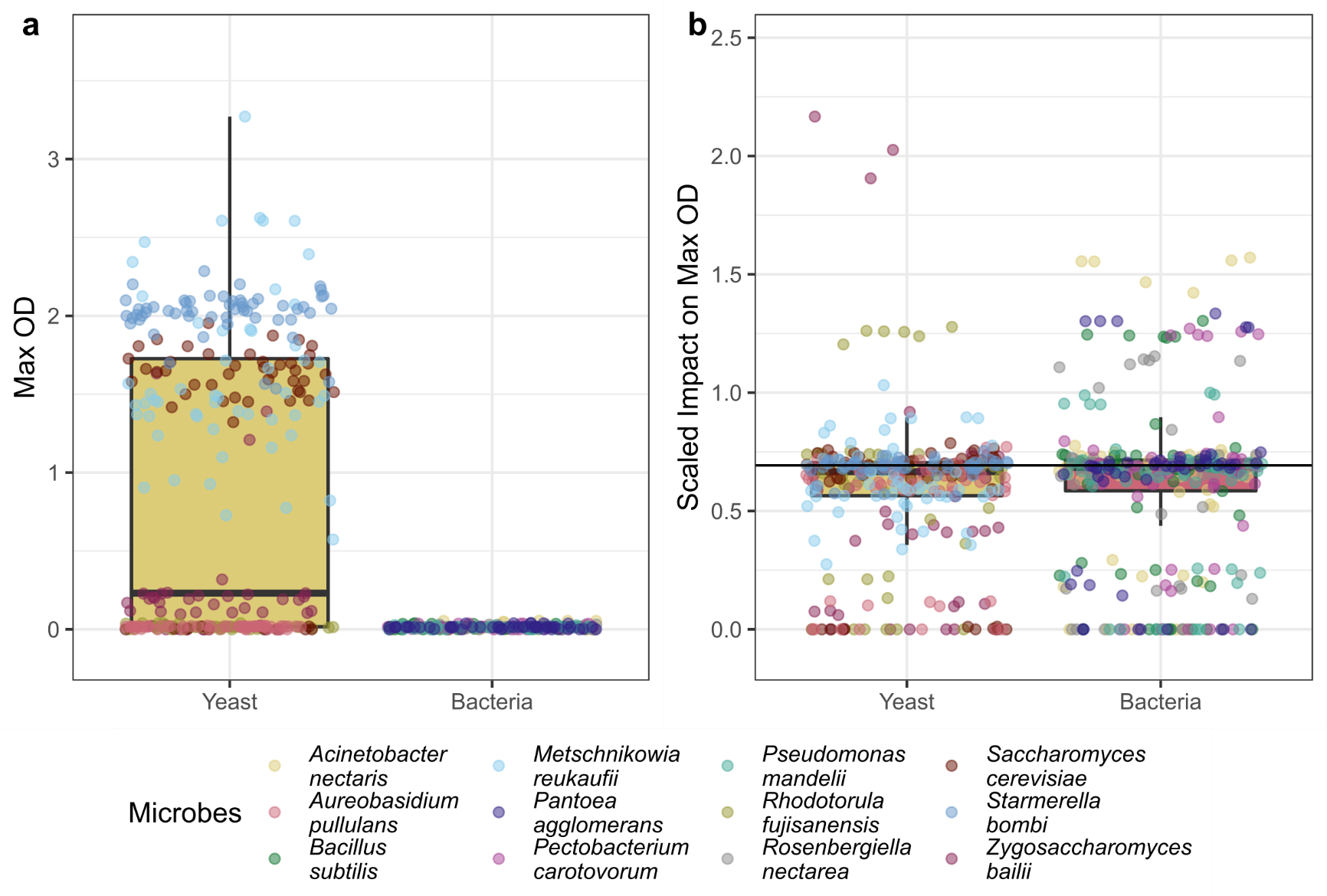


**Supplemental Figure 5.** Bacteria and Yeast differed overall in their maximum OD and in their susceptibility to treatments. The Y axis is the scaled impact of a treatment on maximum OD compared to controls.

**
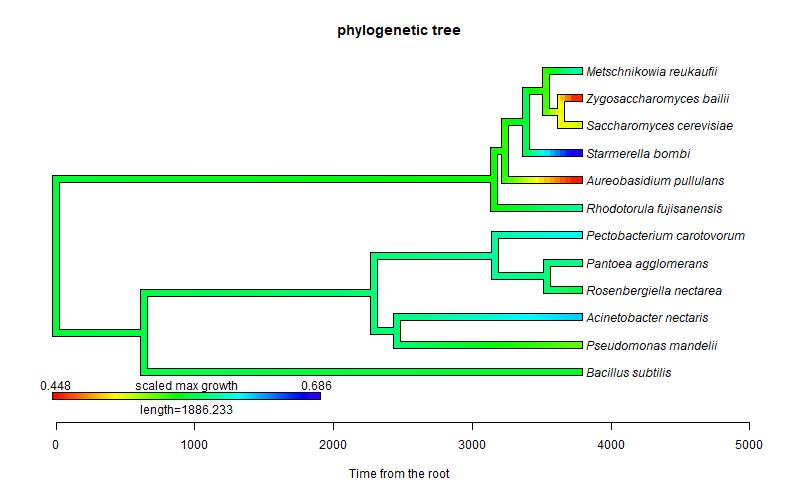
**

**Supplemental Figure 6.** A phylogenetic tree showing the relationship of the assayed microbes with branch tips colored by their scaled max growth average across all treatments, i.e., the overall susceptibility or resistance to nectar compounds. The X axis shows time in millions of years.
